# Rapidly enlarging renal tumor during pregnancy: diagnostic and management dilemma

**DOI:** 10.15586/jkcvhl.2014.6

**Published:** 2014-04-22

**Authors:** Kor Woi Tiang, Keng Lim Ng, Antonio Vega-Vega, Simon Wood

**Affiliations:** 1 The University of Queensland, Brisbane, Australia; 2 Department of Surgery, University Malaya, Kuala Lumpur, Malaysia; 3 Department of Urology, Princess Alexandra Hospital, Brisbane, Australia; 4 Rockhampton Hospital, Rockhampton, Australia

## Abstract

Urological tumors diagnosed during pregnancy are rare. However, the incidence seems to be increasing largely due to advancements in modern imaging techniques and improved antenatal care. The diagnosis and management of renal tumors during pregnancy poses a dilemma to clinicians. This case report highlights the challenges in managing a large chromophobe renal cell carcinoma in a young primigravida patient. Proper antenatal assessment, a multidisciplinary team approach and appropriate discussion with patient are important determinants to achieve the best clinical outcomes for both the mother and the baby.

## Introduction

Pregnancy-associated cancers have increased statistically although it remains rare. The incidence is reported to be one for every 1000 maternities (1). This increase is only partially explained by increasing maternal age. Improved imaging techniques and increased interaction with health services during pregnancy could be the reasons for the detection of higher rates of pregnancy-associated cancers (2) with renal cell carcinoma (RCC) being the most common. In the past, management of intrapartum malignancy has been controversial due to the difficulties in predicting and achieving best maternal-fetal outcomes. However, these have changed recently with improvements in surgical techniques and intensive care facilities. This case report highlights the challenges in managing a large chromophobe RCC in a young primigravida patient.

## Case Report

We report a case of a renal tumor diagnosed during the first trimester in a Gravida 1 Para 0, previously healthy 21-year-old female. The renal tumor was diagnosed incidentally during routine antenatal ultrasound scan. The patient reported vague and mild abdominal discomfort of unknown duration leading up to her pregnancy. The abdominal discomfort was soon attributed by the patient to be pregnancy related. In addition, the patient lived in a regional area with reduced access to tertiary care. Following the initial antenatal scan, which did not reveal any renal mass, another abdominal ultrasound ordered by her General Practitioner confirmed a large left renal upper pole mass. Follow up imaging at 31 weeks gestation revealed a rapidly enlarging renal tumor, with size of 17.0x15.0x10.0cm3. A decision was made by the treating medical team to defer definitive treatment until postpartum.

**Figure 1:**
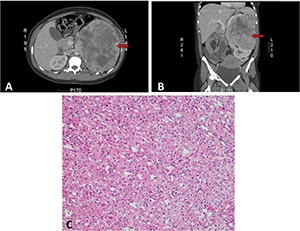
**Computed tomography of abdomen and the histology of RCC.** A, Axial CT scan of left renal tumor occupying almost half of the abdomen (arrow). B, Coronal CT scan view of left renal tumor at the upper pole displacing the normal lower pole renal parenchyma (arrow). C, Hematoxylin and Eosin staining of the tumor showed chomophobe morphology with peri-nuclear halo and transitional cells.

**Figure 2:**
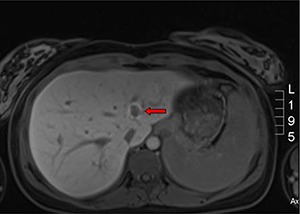
**T1 weighted MRI (axial view):** Liver lesion consistent with focal nodular hyperplasia (arrow).

A healthy baby girl was delivered via spontaneous vaginal delivery at term. Within a few days postpartum, an abdominal computed tomography scan revealed a large left heterogeneous mass with mixed density and contrast enhancement and a small indeterminate liver lesion (Figures 1 A and B). Open left radical nephrectomy was performed 3 weeks following delivery. Intra-operatively, there was no evidence of metastatic disease and a 2086g left renal tumor measuring 23.0x17.0x13.0 cm^3^ was successfully removed. The post-operative recovery was uneventful. Pathological analysis revealed chromophobe RCC, pT2bN0M0 with peri-nuclear halo and transitional cells (Figure 1 C). There was no venous infiltration and the tumor was CK7, CD117 positive and CD10 negative with no sarcomatoid or rhabdoid differentiation. Surgical margins were clear. Magnetic resonance imaging at one week post-operation showed a small liver nodule consistent with focal nodular hyperplasia and no appearance of typical metastasis in the liver (Figure 2). On follow up, the patient remained well with no evidence of recurrence or metastases.

## Discussion

Urological tumors diagnosed during pregnancy are rare. Approximately 70 cases of RCC have been reported so far (3). Of these, the incidence of chromophobe RCC remained the lowest (4). RCC often mimics common benign pregnancy-related symptoms such as abdominal pain, urinary tract infections and urinary calculi. Therefore, similar symptomatology between an enlarging RCC and pregnancy-related conditions may delay the diagnosis and treatment of such renal tumors (5). In the literature, the most common symptoms in pregnant women with renal cancer were: palpable mass (88%), pain (50%), hematuria (47%), fever (21%), hypertension (18%) while the classical triad of symptoms was noted in only 21% of cases (6). In our case, the patient did notice mild abdominal discomfort which was dismissed by the patient as pregnancy-related aches.

It has been proposed that pregnancy related hormonal changes may act as promoters for renal malignancy. For example, high estrogen levels during pregnancy can promote malignant changes by stimulating renal cell proliferation (7). However, there has been no demonstrable immunodeficiency in pregnancy to antigens carried by tumor cells (8). Furthermore, in most cases, the biological behavior of malignancy is not influenced by pregnancy (9).

The safest imaging modalities in pregnancy are ultrasonography and Magnetic Resonance Imaging (MRI) with both of them containing minimal risk for ionizing radiations. However, several factors may explain the delay in diagnosis of renal cancer in pregnancy. Most small renal masses are asymptomatic and further assessment is often not indicated largely because of insufficient safety data of radiation during pregnancy. (10). Radiological assessment of extra pelvic organs is also not part of routine antenatal screen. The early pregnancy scan usually performed before 15 weeks establishes the viability, gestational age, fetal number and gross anomalies. The anomaly scan usually performed at 20 weeks involves assessment of major organs and seek out any anomalies. These recommended antenatal scans usually focus only on the pelvic region and fetal structures.

A full abdominal ultrasound during pregnancy will allow the early diagnosis of malignancies or diseases of other organs which may impact upon the well-being of the mother and fetus. This slight modification of full abdominal ultrasound is fast, simple to perform, and safe for both fetus and mother. Some authors recommend that a total abdominal ultrasound be performed at least once in all pregnancies in addition to routine fetal ultrasonography, especially in the presence of loin or abdominal pain, hematuria or hypertension (11, 12). Once a urological malignancy is detected on ultrasonography, the safest modality for further imaging studies is MRI. Radiological contrast agents should be avoided due to transplacental risk and teratogenic effects (13). In our case, serial ultrasound scans were used as MRI was not readily accessible in the regional center.

In the case of RCC, timely diagnosis and management is crucial in predicting maternal-fetal prognosis. Timing of surgery for renal tumors during pregnancy is controversial. Recommendations regarding the timing of surgery are dictated by time of diagnosis, size and stage of tumor, probability of malignancy, general health of the mother and probability of survival of the fetus. Probability of cure is directly related to the stage of malignancy. Surgical resection remains the mainstay of treatment. Most renal neoplasms are slow growing with an average volume doubling time of more than 500 days (4).Therefore it may be reasonable to await fetal delivery prior to surgery if the neoplasm is detected during late stages of pregnancy. During diagnosis of suspicious malignant renal mass in the first trimester, surgery is often not delayed despite the small increased risk of miscarriage (14). However, surgery during second trimester will need to balance the maternal and fetal risk of abortion and preterm labor with the risks of delaying surgery. Avoidance of disruption of the peritoneal cavity in the extra-peritoneal approach may theoretically be associated with less uterine irritation and in turn fewer obstetrics complications, including preterm labor (15). This is especially critical in the management of small renal cancers due to increased risk of maternal-fetal adverse outcome before 32 weeks gestation (16).

With the discovery of renal tumor during third trimester, delivery followed by nephrectomy is recommended (17). It is reasonably safe to observe small renal masses and a short delay in definitive treatment in the third trimester. On the other hand, for large tumors, or tumors which exhibit very rapid growth as occurred in this case, nephrectomy should not be delayed. If the diagnosis of renal tumor is near term, nephrectomy should be postponed till post-delivery. However, some physicians recommend immediate nephrectomy irrespective of the pregnancy stage. Because, they feel that the main priority is to the mother’s welfare (18). Nevertheless, in the presence of malignancy, maternal-fetal morbidity and mortality should be carefully considered with maternal health as the top priority (19). The patient needs to be thoroughly informed of all possible risks versus benefits of management options laid out through discussions in a multi-disciplinary conference (urologists, obstetricians and oncologists).

## Conclusion

Routine complete abdominal ultrasound screening during pregnancy will benefit patients. Furthermore, a patient-centered multidisciplinary approach is preferred to maximize a favorable maternal-fetal outcome in the management of suspicious renal masses during pregnancy. Prompt diagnosis and management of RCC during pregnancy is important because it is potentially curable.
